# Associations between Healthy Lifestyle and All-Cause Mortality in Individuals with Metabolic Associated Fatty Liver Disease

**DOI:** 10.3390/nu14204222

**Published:** 2022-10-11

**Authors:** Xinyu Wang, Aruna Wang, Ruosu Zhang, Si Cheng, Yuanjie Pang

**Affiliations:** Department of Epidemiology & Biostatistics, School of Public Health, Peking University, 38 Xueyuan Road, Beijing 100191, China

**Keywords:** healthy lifestyle, fatty liver disease, all-cause mortality, advanced fibrosis, liver enzyme

## Abstract

Background and Aims: There is limited evidence about the association of healthy lifestyle and all-cause mortality in individuals with metabolic associated fatty liver disease (MAFLD). We aimed to examine this association and compare it with the association in those without MAFLD. Methods: A prospective cohort study was performed and linked mortality data through 2019 in the National Health Nutrition Examination Survey (NHANES 1999–2010). A healthy lifestyle score was constructed from cigarette smoking, alcohol drinking, healthy eating score, and leisure-time physical activity. Risk stratification was conducted in participants with MAFLD by fibrosis biomarkers and liver enzymes. Survey-weight adjusted Cox regression was used to estimate adjusted hazard ratios (HRs) and confidence intervals (CIs) for all-cause mortality associated with healthy lifestyle. Results: There was a protective association between healthy lifestyle and all-cause mortality in participants with MAFLD (HR per 1-unit increase of healthy lifestyle score 0.77 [95% CI 0.69–0.85]), with no difference from the association in participants without MAFLD (HR 0.77 [0.72–0.82]). In participants with MAFLD, the associations tended to be stronger in those with lower risk of advanced fibrosis (HR per 1-unit increase of healthy lifestyle score 0.64 [0.50–0.79] for low NAFLD fibrosis score [NFS] and 0.84 [0.75–0.93] for high NFS, *p*-value for interaction 0.02), but did not differ by liver enzymes. The results for non-alcoholic fatty liver disease (NAFLD) mirrored those for MAFLD. Conclusions: Healthy lifestyle showed protective associations with all-cause mortality regardless of MAFLD status, and the associations tended to be stronger in those with lower risk of advanced fibrosis. Timely lifestyle modification matters for individuals with MAFLD.

## 1. Introduction

Metabolic associated fatty liver disease (MAFLD), an updated nomenclature of non-alcoholic fatty liver disease (NAFLD), was proposed to reflect current knowledge of fatty liver disease associated with metabolic dysfunction [1]. The pooled global prevalence of NAFLD and MAFLD was reported to be 29.8% and 38.8%, respectively [2,3]. In the United States (US), the prevalence of MAFLD increased from 34.4% in 2011 to 38.1% in 2018, and its close relationship with overall mortality led to concerns about how to improve the prognosis of patients with MAFLD [4,5]. In addition, since advanced liver fibrosis is a key prognostic marker for overall mortality among patients with NAFLD, non-invasive biomarkers may help to identify advanced fibrosis in individuals with MAFLD for early intervention [6,7].

Currently, there is no approved therapy for NAFLD, and lifestyle modification remains a key treatment [8]. Adopting a healthy lifestyle (including not smoking or drinking, eating healthily, and taking adequate physical activity) is associated with lower risks of all-cause and cause-specific mortality in the general population [9,10,11]. While prior studies have assessed the relationships between individual healthy lifestyle component and mortality in patients with NAFLD, there is little evidence on the associations of a combined healthy lifestyle with all-cause mortality in individuals with fatty liver, especially in those with the newly defined MAFLD [12,13,14,15,16]. Examining these associations may help to quantify the extent to which lifestyle modification can prevent mortality in individuals with MAFLD and assess whether individuals with MAFLD may achieve similar benefits through lifestyle modification compared with those without.

Therefore, using a nationally representative sample of US adults, we aimed to (1) examine the associations between healthy lifestyle and all-cause mortality in participants with MAFLD and compare them with the associations in those without MAFLD; (2) examine the associations between healthy lifestyle and all-cause mortality in participants with MAFLD stratified by levels of fibrosis or liver enzymes; and (3) examine whether similar patterns of associations were observed in NAFLD participants.

## 2. Methods

### 2.1. Study Population

This study was based on the National Health Nutrition Examination Survey (NHANES) conducted by the National Center for Health Statistics, Centers for Disease Control and Prevention. NHANES is a nationally representative cross-sectional survey of the civilian non-institutionalized US population, with the use of a complex, multistage, stratified sampling design. Details of the study design and data collection have been previously described [17]. The National Center for Health Statistics Research Ethics Review Board approved the NHANES protocol and informed consent was obtained from all subjects.

The current study was based on an analysis of data from the combined 6 continuous NHANES survey cycles (1999–2010). Overall, 31,427 participants who were aged 20 years and older and not pregnant at baseline were included. Those with missing information on mortality status (n = 46), lifestyle factors (n = 13,939), and MAFLD or NAFLD status (n = 9530) were further excluded, leaving 7912 individuals for the final analysis.

### 2.2. Definition of Lifestyle Factors

We constructed a healthy lifestyle score including cigarette smoking, alcohol consumption, physical activity, and diet according to a previous NHANES study [9]. All lifestyle factors were obtained through structured questionnaires and 24-hour dietary recalls. Never smoking was considered as a healthy level, which was defined in the questionnaire as smoking fewer than 100 cigarettes in life. Frequency and volume of current alcohol consumption were self-reported, and a healthy level was defined as daily consumption of ≤1 drink for women and ≤2 drinks for men, according to the dietary guidelines in the US [18]. For physical activity, weekly metabolic equivalent hours of leisure-time physical activity were calculated and categorized into tertiles, and the top tertile was defined as a healthy level of physical activity. Dietary quality was assessed by healthy eating index (HEI) 2015 scores, which aligns with the 2020–2025 Dietary Guidelines for Americans [18,19]. A healthy diet was defined as the HEI score in the top two quintiles of distribution. Table S1 provides details of HEI-2015 construction.

For each lifestyle factor, we assigned 1 point to a healthy level and 0 points to an unhealthy level. Therefore, the healthy lifestyle score was the sum of the points and ranged between 0 and 4, with a higher score indicating a healthier lifestyle.

### 2.3. Clinical and Laboratory Evaluations

Hypertension was identified as mean systolic blood pressure ≥130 mmHg or diastolic blood pressure ≥85 mmHg and/or currently taking anti-hypertensive medication. Diabetes was defined as having either diagnosed diabetes, fasting glucose ≥7.0 mmol/L, hemoglobin A1c ≥ 48 mmol/mol, and/or treatment with anti-diabetic medication.

NAFLD was determined by US fatty liver index (FLI) ≥30 in the absence of excessive alcohol consumption (>21 drinks/week in men and >14 drinks/week in women) and viral hepatitis (positive serum hepatitis C antibody and/or positive serum hepatitis B surface antigen) [5]. US FLI is an improved FLI for the multiethnic US population and used as a surrogate for the clinical diagnosis of NAFLD because ultrasonography data are not available for the current survey cycles [20].

MAFLD was diagnosed by US FLI ≥ 30 with ≥1 of the following: (i) overweight or obese (body mass index [BMI] ≥ 25 kg/m^2^), (ii) diabetes mellitus, (iii) at least 2 metabolic risk abnormalities. Metabolic risk abnormalities consisted of (i) waist circumference (WC) ≥102 cm for men or ≥88 cm for women, (ii) blood pressure ≥ 130/85 mmHg or specific drug treatment, (iii) fasting plasma triglycerides ≥1.70 mmol/L or specific drug treatment, (iv) plasma high-density lipoprotein (HDL) cholesterol <1.0 mmol/L for men or <1.3 mmol/L for women or specific drug treatment, (v) prediabetes (fasting glucose 5.6–6.9 mmol/L or hemoglobin A1c 39–47 mmol/mol), (vi) homeostasis model assessment of insulin resistance (HOMA-IR) score ≥ 2.5, (vii) plasma high-sensitivity C-reactive protein (CRP) level > 2 mg/L [1].

Regarding advanced fibrosis, we calculated NAFLD fibrosis score (NFS), fibrosis-4 (FIB-4) score, and Forn’s score based on published equations, with the cut-offs of −1.455, 1.30, and 4.21 to categorize participants with MAFLD/NAFLD into 2 groups [21,22,23]. In addition, participants with MAFLD/NAFLD were grouped by levels of liver enzymes, known as risk factors for fatty liver progression. Elevated alanine aminotransferase (ALT) was defined as >40 IU/L in men or >31 IU/L in women, and elevated γ-glutamyl transferase (GGT) as >51 IU/L in men or >33 IU/L in women based on previous NHANES studies [24,25].

### 2.4. Ascertainment of Death

All-cause mortality data were acquired from the open-source linkage data from National Death Index (NDI) [26]. Individuals aged 18 years and above with sufficient identifying data were eligible for follow-up, and those who did not have any death records were presumed alive through the follow-up period. Follow-up time was calculated from the date of examination at the mobile examination center to date of death or 31 December 2019, whichever came first. Causes of death were categorized according to the Underlying Cause of Death 113 (UCOD_113) code, which was classified based on the International Classification of Diseases, 10th edition (ICD-10). Cause-specific mortalities were assessed as cardiovascular disease (UCOD_113 code: 054–068) and cancer (019–043).

### 2.5. Statistical Analysis

To estimate appropriate variance and statistics representative of US adults, we analyzed the data using appropriate sampling weights according to the NHANES statistical analysis guideline [27]. The weight-adjusted means ± standard errors of continuous variables and weight-adjusted proportions of categorical variables are presented among comparison groups. We compared baseline characteristics using a *t*-statistic for continuous variables and the chi-square test for categorical variables, all adjusted for sampling weights.

Survey-weight adjusted multivariable Cox proportional hazards regression models were used to calculate hazard ratios (HRs) of mortality and 95% confidence intervals (CIs) after consideration of potential demographic and clinical confounders. Model 1 was adjusted for age, gender, ethnicity, marital status, and education. Model 2 was adjusted for BMI, WC, CRP, HDL, hypertension, and diabetes in addition to model 1.

Analyses were performed using R (version 4.2.1; R Foundation for Statistical Computing, Vienna, Austria). The HEI-2015 scores were calculated using SAS version 9.4 (SAS Institute, Cary, NC, USA).

## 3. Results

### 3.1. Population Characteristics

A total of 2616 and 2446 participants out of 7912 were diagnosed as MAFLD and NAFLD, respectively. Participants with MAFLD were older and more likely to be male and obese than those without MAFLD (Table 1). The laboratory parameters significantly differed between the two groups. Cases with MAFLD had higher serum levels of lipids, glucose, and liver enzymes. Participants without MAFLD tended to smoke less, eat healthier, and exercise more than those with MAFLD. Over half of cases with MAFLD had hypertension, whereas this proportion was 31.4% in the non-MAFLD group. Characteristics of participants with NAFLD were similar to those with MAFLD.

### 3.2. Associations of Healthy Lifestyle with All-Cause Mortality in Participants with MAFLD

During a median follow-up of 14.6 (interquartile range: 11.9–17.8) years, there were a total of 1820 deaths. In participants with MAFLD, compared with those with a healthy lifestyle score of 0 or 1, those with a healthy lifestyle score of 2, 3, and 4 had a multivariate-adjusted HR of 0.70 (0.56–0.87), 0.67 (0.53–0.85), and 0.29 (0.18–0.47), respectively. The corresponding HRs for participants without MAFLD were 0.76 (95% CI 0.62–0.92), 0.62 (0.52–0.74), and 0.39 (0.30–0.52). The protective associations between healthy lifestyle and all-cause mortality did not differ by MAFLD status (*p*-value for interaction 0.83–0.96, Table 2). Associations between individual healthy lifestyle and all-cause mortality in participants with and without MAFLD are shown in Table S7.

When participants with MAFLD were stratified by demographic factors, the protective associations attenuated in participants aged ≥50 years, but did not differ by gender or ethnicity (Table S2). The adjusted HR per 1-unit increase in healthy lifestyle score was 0.84 (0.75–0.94) in participants aged ≥50 and 0.40 (0.29–0.55) in those aged <50 years (*p*-value for interaction <0.001).

### 3.3. Associations of Healthy Lifestyle with All-Cause Mortality in Participants with MAFLD by Liver Fibrosis Status and Liver Enzymes

Adopting a healthy lifestyle showed a weaker protective association with all-cause mortality in participants with advanced fibrosis than those without (*p*-value for interaction 0.01–0.16, Table 3). The adjusted HR per 1-unit increase of lifestyle score was 0.63 (0.50–0.79) and 0.84 (0.75–0.93) in participants with low and high NFS score, respectively. The corresponding HRs using FIB-4 and Forn’s score were 0.71 (0.60–0.83) and 0.82 (0.71–0.95), and 0.64 (0.50–0.82) and 0.84 (0.75–0.94), respectively. Associations for individual healthy lifestyle and all-cause mortality are shown in Table S8.

Serum levels of liver enzymes did not interact with healthy lifestyle on all-cause mortality in participants with MAFLD (*p*-value for interaction 0.62–0.98, Table 4). The adjusted HR per 1-unit increase of lifestyle score in participants with elevated GGT was 0.80 (0.68–0.93), and those with normal levels of GGT had an adjusted HR of 0.77 (0.68–0.87). For ALT, the corresponding HRs were 0.71 (0.53–0.94) and 0.80 (0.72–0.89) for elevated and normal levels, respectively.

### 3.4. Associations of Healthy Lifestyle with All-Cause Mortality by NAFLD Status

When stratified by NAFLD status, patterns of associations were mostly similar to those observed for MAFLD. The protective associations did not differ by NAFLD status or serum levels of liver enzymes in participants with NAFLD (Tables S4 and S6). However, the associations of healthy lifestyle with all-cause mortality differed when stratified by status of advanced fibrosis (*p*-value for interaction 0.01–0.08, Table S5). Associations for individual healthy lifestyle and all-cause mortality are shown in Tables S7 and S9.

## 4. Discussion

In the current study, we found a protective association of healthy lifestyle with all-cause mortality in individuals with MAFLD, and the protective association was stronger in participants with lower risk of advanced fibrosis. Results for NAFLD were mostly similar. These findings suggest that a healthy lifestyle provides a similar survival benefit in adults with MAFLD compared with those without, and individuals with lower risk of advanced fibrosis may benefit more from adopting a healthy lifestyle.

Our results were generally consistent with previous studies examining the associations of combined healthy lifestyle with all-cause mortality in the general population [9,10,11]. In a meta-analysis including 74 studies and 2,584,766 participants, participants with the healthiest lifestyle had a 55% (HR 0.45 [0.41–0.48], between-study heterogeneity *I*^2^ = 91.0%) lower risk of all-cause mortality compared with those with the unhealthiest lifestyle [11]. In studies conducted in North America, the corresponding HR and *I*^2^ were 0.49 (0.44–0.55) and 93.1%, respectively. Although heterogeneity between studies was large due to the different definitions of healthy lifestyle in individual studies, our point estimates were generally consistent with this meta-analysis (HR 0.29 [0.18–0.47] and 0.39 [0.30–0.52] for participants with and without MAFLD, respectively). In another prospective cohort study in NHANES (1988–2014), the HR per 1-unit increase in healthy lifestyle score was 0.83 (0.80–0.85), which was slightly weaker than the estimate in the current study [9]. The difference may result from the different standards of the diet score and definitions of physical activity in NHANES III (1988–1994), as well as different adjustments in regression models. The current study expanded the literature by assessing the beneficial effects of a healthy lifestyle in subpopulations with liver steatosis and fibrosis.

To the best of our knowledge, this is the first study examining the associations of a combined healthy lifestyle with all-cause mortality in participants with MAFLD. Previous studies mainly focused on the protective associations of healthy lifestyle with death in patients with NAFLD. A prospective cohort study in NHANES reported an inverse association between the number of cardiovascular health metrics (i.e., BMI, smoking status, physical activity, diet score, blood pressure, total cholesterol, and glycemic control) and risk of all-cause mortality in participants with NAFLD, which is consistent with our study [14]. Compared with the cardiovascular health metrics, our definition of healthy lifestyle was more easily achievable in the general population. Another prospective cohort study in NHANES reported a protective association of healthy diet with all-cause mortality in subjects without NAFLD, but not in those with NAFLD [16]. In contrast, our study observed null associations for healthy diet in participants with and without NAFLD (HR 0.89 [0.72–1.09] and 0.86 [0.72–1.03] respectively, Table S7), which may result from different calculations for HEI. In support of our study, several prospective cohort studies reported associations of smoking, drinking, and physical inactivity with all-cause mortality in patients with NAFLD [12,13,15,28]. Our study expanded the literature and found different magnitudes of associations across subpopulations with varying risk of steatosis and advanced fibrosis.

The current study demonstrated that, in participants with MAFLD, the protective associations of healthy lifestyle with all-cause mortality were weaker in those with higher risk of advanced fibrosis, but did not differ by liver enzymes. Subclinical liver disease might be accompanied with elevated levels of fibrosis biomarkers, which might progress to clinical liver disease during the follow-up period. Participants with progressive symptoms might change their lifestyles, such as reducing physical activity and changing dietary patterns. However, lifestyle information was collected only at baseline in the current study, which might attenuate the protective associations. 

Consistent with previous studies [29,30], additional adjustment for BMI, WC, CRP, and other biochemical factors did not attenuate the protective association between healthy lifestyle and all-cause mortality. It is possible that there are other pathways through which healthy lifestyle can improve the prognosis of fatty liver. Moreover, previous meta-analyses of randomized controlled trials (RCTs) have shown that increased exercise and improved diet can prevent NAFLD progression (assessed by degrees of hepatic steatosis and progressive liver diseases) through weight loss or anti-inflammatory effects, suggesting that obesity and inflammation might be potential mediators between healthy lifestyle and liver-related mortality [31,32,33]. However, liver-related mortality accounted for a small proportion of all-cause mortality and we could not retrieve data of cause-specific mortality due to data restrictions.

Our findings have several clinical implications. The results demonstrate protective associations of healthy lifestyle with all-cause mortality regardless of MAFLD status. This finding supports expert guidelines that lifestyle modification should be the cornerstone for the management of NAFLD and suggests that similar recommendations should be made for MAFLD. Additionally, adopting a healthy lifestyle may be more effective for MAFLD patients with lower risk of advanced fibrosis, suggesting the importance of timely intervention. 

The strengths of the current study included a comprehensive score for healthy lifestyle, detailed assessments of liver status, a sufficient follow-up period for mortality, high-quality clinical and metabolic variables, and a representative sample of the US population. However, several limitations must be stated. First, hepatic steatosis status was assessed using US FLI and fibrosis status was derived from blood-based biomarkers. However, a recent cross-sectional study in NHANES reported that US FLI showed good diagnostic performance against vibration-controlled transient elastography (positive predictive value 90%, specificity 63.7%) [34]. In addition, biomarkers of advanced fibrosis (e.g., NFS, FIB-4, Forn’s score) have been externally validated and accepted by clinical practice guidelines [35,36]. The summary sensitivities and specificities of NFS (threshold of −1.455) and FIB-4 (threshold of 1.30) reported by previous meta-analyses for detecting advanced fibrosis were 0.72 and 0.70, and 0.84 and 0.89, respectively [37,38]. Second, the healthy lifestyle score was constructed using self-reported information assessed cross-sectionally rather than by dynamic monitoring. Third, due to data restrictions, we were unable to retrieve a detailed ICD-10 classification for cause of death. Instead, we used the UCOD_113 code (a variable to recode all deaths into comparable ICD-10-based underlying cause of death groups), which might lead to misclassification and thus attenuate the associations towards the null (Table S4) [26].

In conclusion, a healthy lifestyle was inversely associated with lower all-cause mortality in individuals with MAFLD, and the associations tended to be stronger in those with lower risk of advanced fibrosis. The American Gastroenterological Association recommends lifestyle modification involving diet and exercise to achieve weight loss in the management of NAFLD [32]. The current study suggests that a similar strategy should be adopted for MAFLD. Future studies are warranted to assess the benefits of comprehensive lifestyle interventions in the management of MAFLD.

## Figures and Tables

**Table 1:** Baseline characteristics by MAFLD and NAFLD status.

	Non-NAFLD (n = 5466)	NAFLD (n = 2446)	*p*-Value	Non-MAFLD (n = 5296)	MAFLD (n = 2616)	*p*-Value
Demographic information						
Age (SD), years	44.8 (0.4)	51.5 (0.6)	<0.001	44.6 (0.4)	51.5 (0.5)	<0.001
Male, %	54.9	57.4	<0.001	44.2	58.7	<0.001
Ethnicity, %			0.430			0.415
Non-Hispanic white	74.3	75.6		74.3	75.5	
Non-Hispanic black	12.0	6.0		12.1	6.1	
Hispanic	4.1	4.8		4.2	4.7	
Others	9.6	13.6		9.5	13.7	
Education, %			0.024			0.006
Less than high school	12.6	16.2		12.4	16.5	
High school	19.7	20.2		19.5	20.8	
Above high school	67.6	63.6		68.1	62.7	
Ever married, %	71.0	82.5	<0.001	70.9	82.0	<0.001
Physical examination and laboratory analysis						
Body mass index (SD), kg/m^2^	26.1 (0.1)	33.4 (0.2)	<0.001	25.9 (0.1)	33.2 (0.2)	<0.001
Waist circumference (SD), cm	91.0 (0.2)	111.4 (0.5)	<0.001	90.5 (0.2)	111.3 (0.5)	<0.001
Total cholesterol (SD), mmol/L	5.1 (0.02)	5.3 (0.04)	<0.001	5.1 (0.02)	5.4 (0.04)	<0.001
HDL-cholesterol (SD), mmol/L	1.4 (0.01)	1.2 (0.01)	<0.001	1.5 (0.01)	1.2 (0.01)	<0.001
CRP (SD), mg/L	3.4 (0.2)	6.2 (0.3)	<0.001	3.3 (0.2)	6.2 (0.3)	<0.001
Triglyceride (SD), mmol/L	1.4 (0.02)	2.3 (0.1)	<0.001	1.3 (0.02)	2.3 (0.1)	<0.001
Fasting glucose (SD), mmol/L	5.4 (0.03)	6.5 (0.1)	<0.001	5.4 (0.02)	6.5 (0.1)	<0.001
Hemoglobin A1c (SD), mmol/mol	34.6 (0.2)	40.4 (0.3)	<0.001	34.5 (0.2)	40.4 (0.3)	<0.001
ALT (SD), IU/L	23.4 (0.3)	32.9 (0.6)	<0.001	22.5 (0.3)	34.4 (0.7)	<0.001
AST (SD), IU/L	24.3 (0.3)	27.4 (0.4)	<0.001	23.8 (0.3)	28.4 (0.6)	<0.001
GGT (SD), IU/L	24.2 (0.6)	43.1 (1.6)	<0.001	21.9 (0.4)	47.3 (1.9)	<0.001
Lifestyle and diseases						
Never smoking, %	51.8	48.7	0.057	52.8	46.5	<0.001
Healthy drinking, %	88.5	96.8	<0.001	90.5	91.6	0.191
HEI-2015 score (SD)	49.7 (0.4)	48.2 (0.4)	0.002	49.7 (0.4)	48.4 (0.4)	0.004
LTPA (SD), MET-h/week	22.4 (1.0)	12.4 (0.7)	<0.001	22.6 (1.1)	12.6 (0.7)	<0.001
Hypertension, %	32.6	59.0	<0.001	31.4	59.9	<0.001
Diabetes, %	5.0	22.4	<0.001	4.4	22.6	<0.001

Data are shown as the weighted mean ± standard errors or weighted frequency as appropriate. Survey-weight adjusted t-test and the chi-square test for categorical variables were used in this analysis. ALT, alanine aminotransferase; AST, aspartate aminotransferase; CRP, C-reactive protein; GGT, γ-glutamyl transferase; HDL, high-density lipoprotein; HEI, healthy eating index; LTPA, leisure-time physical activity; MAFLD, metabolic associated fatty liver disease; NAFLD, non-alcoholic fatty liver disease; SD, standard deviation.

**Table 2:** Associations between healthy lifestyle and all-cause mortality in participants with and without MAFLD.

	No. Death/No. Participants	Model 1	Model 2
		HR (95% CI)	HR (95% CI)
Non-MAFLD (n = 5296)			
Score 0/1	301/1217	1.00	1.00
Score 2	365/1939	0.73 (0.59, 0.89)	0.76 (0.62, 0.92)
Score 3	308/1639	0.59 (0.49, 0.70)	0.62 (0.52, 0.74)
Score 4	60/501	0.36 (0.27, 0.49)	0.39 (0.30, 0.52)
Per 1-unit increase		0.75 (0.70, 0.80)	0.77 (0.72, 0.82)
MAFLD (n = 2616)			
Score 0/1	269/753	1.00	1.00
Score 2	295/1037	0.67 (0.54, 0.83)	0.70 (0.56, 0.87)
Score 3	199/694	0.65 (0.52, 0.82)	0.67 (0.53, 0.85)
Score 4	23/132	0.27 (0.17, 0.43)	0.29 (0.18, 0.47)
Per 1-unit increase		0.76 (0.69, 0.84)	0.77 (0.69, 0.85)
*p*-value for interaction		0.83	0.96

Survey-weight adjusted multivariable Cox proportional models were used in this analysis. Model 1 was adjusted for age, gender, ethnicity, education, and marital status. Model 2 was adjusted for BMI, WC, CRP, TG, HDL, hypertension, and diabetes in addition to model 1. CI, confidence interval; HR, hazards ratio; MAFLD, metabolic associated fatty liver disease.

**Table 3:** Associations between healthy lifestyle and all-cause mortality in MAFLD participants with and without advanced fibrosis.

	No. Death/No. Participants	Model 1	Model 2
		HR (95% CI)	HR (95% CI)
NFS (n = 2576)			
Low NFS (n = 1348)			
Score 0/1	89/397	1.00	1.00
Score 2	58/516	0.44 (0.25, 0.77)	0.42 (0.25, 0.69)
Score 3	52/371	0.53 (0.32, 0.87)	0.47 (0.28, 0.77)
Score 4	6/64	0.20 (0.07, 0.52)	0.20 (0.07, 0.54)
Per 1-unit increase		0.65 (0.51, 0.85)	0.63 (0.50, 0.79)
High NFS (n = 1228)			
Score 0/1	175/350	1.00	1.00
Score 2	230/505	0.84 (0.66, 1.07)	0.90 (0.69, 1.16)
Score 3	140/308	0.76 (0.58, 1.00)	0.80 (0.59, 1.07)
Score 4	16/65	0.32 (0.19, 0.54)	0.37 (0.21, 0.63)
Per 1-unit increase		0.82 (0.75, 0.90)	0.84 (0.75, 0.93)
*p-*value for interaction		0.05	0.02
FIB-4 (n = 2604)			
Low FIB-4 (n = 1773)			
Score 0/1	135/537	1.00	1.00
Score 2	115/685	0.62 (0.44, 0.86)	0.65 (0.47, 0.91)
Score 3	75/470	0.57 (0.38, 0.85)	0.56 (0.37, 0.84)
Score 4	9/81	0.23 (0.11, 0.46)	0.21 (0.09, 0.45)
Per 1-unit increase		0.71 (0.60, 0.84)	0.71 (0.60, 0.83)
High FIB-4 (n = 831)			
Score 0/1	134/216	1.00	1.00
Score 2	180/346	0.71 (0.51, 0.98)	0.73 (0.53, 1.01)
Score 3	124/220	0.71 (0.50, 1.01)	0.75 (0.52, 1.08)
Score 4	14/49	0.31 (0.15, 0.63)	0.37 (0.19, 0.75)
Per 1-unit increase		0.80 (0.69, 0.92)	0.82 (0.71, 0.95)
*p-*value for interaction		0.16	0.10
Forn’s score (n = 2608)			
Low Forn’s score (n = 1384)			
Score 0/1	76/400	1.00	1.00
Score 2	64/537	0.61 (0.38, 0.92)	0.61 (0.37, 0.99)
Score 3	51/383	0.49 (0.28, 0.85)	0.47 (0.26, 0.83)
Score 4	4/64	0.15 (0.05, 0.41)	0.13 (0.05, 0.40)
Per 1-unit increase		0.65 (0.50, 0.83)	0.64 (0.50, 0.82)
High Forn’s score (n = 1224)			
Score 0/1	193/353	1.00	1.00
Score 2	231/499	0.70 (0.55, 0.89)	0.73 (0.57, 0.93)
Score 3	148/306	0.72 (0.55, 0.95)	0.76 (0.57, 1.02)
Score 4	19/66	0.33 (0.20, 0.57)	0.39 (0.23, 0.67)
Per 1-unit increase		0.82 (0.73, 0.91)	0.84 (0.75, 0.94)
*p-*value for interaction		0.03	0.01

Survey-weight adjusted multivariable Cox proportional models were used in this analysis. Model 1 was adjusted for age, gender, ethnicity, education, and marital status. Model 2 was adjusted for BMI, WC, CRP, TG, HDL, hypertension, and diabetes in addition to model 1. Cut-off values for fibrosis biomarkers: NFS, −1.455; FIB-4, 1.30; Forn’s score, 4.21 CI, confidence interval; FIB-4, fibrosis-4; HR, hazards ratio; NFS, NAFLD fibrosis score; MAFLD, metabolic associated fatty liver disease.

**Table 4:** Associations between healthy lifestyle and all-cause mortality in MAFLD participants with and without elevated liver enzyme.

	No. Death/No. Participants	Model 1	Model 2
		HR (95% CI)	HR (95% CI)
ALT (n = 2611)			
Normal ALT (n = 1924)			
Score 0/1	207/528	1.00	1.00
Score 2	261/796	0.78 (0.63, 0.97)	0.81 (0.65, 1.00)
Score 3	165/502	0.70 (0.55, 0.90)	0.73 (0.57, 0.94)
Score 4	18/98	0.25 (0.17, 0.38)	0.28 (0.18, 0.43)
Per 1-unit increase		0.79 (0.71, 0.87)	0.80 (0.72, 0.89)
Elevated ALT (n = 687)			
Score 0/1	62/225	1.00	1.00
Score 2	34/236	0.44 (0.23, 0.82)	0.50 (0.25, 0.98)
Score 3	34/192	0.56 (0.30, 1.04)	0.56 (0.29, 1.07)
Score 4	5/34	0.47 (0.14, 1.54)	0.57 (0.15, 2.09)
Per 1-unit increase		0.72 (0.52, 0.98)	0.71 (0.53, 0.94)
*p*-value for interaction		0.98	0.85
GGT (n = 2616)			
Normal GGT (n = 1585)			
Score 0/1	144/401	1.00	1.00
Score 2	189/654	0.72 (0.54, 0.95)	0.77 (0.56, 1.06)
Score 3	131/441	0.59 (0.45, 0.77)	0.65 (0.48, 0.88)
Score 4	14/89	0.23 (0.12, 0.45)	0.29 (0.15, 0.57)
Per 1-unit increase		0.74 (0.66, 0.83)	0.77 (0.68, 0.87)
Elevated GGT (n = 1031)			
Score 0/1	125/352	1.00	1.00
Score 2	106/383	0.61 (0.43, 0.86)	0.61 (0.43, 0.85)
Score 3	68/253	0.79 (0.55, 1.13)	0.79 (0.56, 1.13)
Score 4	9/43	0.40 (0.20, 0.79)	0.40 (0.19, 0.85)
Per 1-unit increase		0.81 (0.69, 0.95)	0.80 (0.68, 0.93)
*p-*value for interaction		0.62	0.62

Survey-weight adjusted multivariable Cox proportional models were used in this analysis. Model 1 was adjusted for age, gender, ethnicity, education, and marital status. Model 2 was adjusted for BMI, WC, CRP, TG, HDL, hypertension, and diabetes in addition to model 1. Cut-off values for liver enzymes: ALT, 40/31 IU/L for men/women; GGT, 51/33 IU/L for men/women. ALT, alanine aminotransferase; AST, aspartate aminotransferase; CI, confidence interval; GGT, γ-glutamyl transferase; HR, hazards ratio; MAFLD, metabolic associated fatty liver disease.

## Data Availability

The datasets used in the current study are publicly available at https://wwwn.cdc.gov/nchs/nhanes.

## Supplementary Materials

The following supporting information can be downloaded at: https://www.mdpi.com/article/10.3390/nu14204222/s1, Table S1: HEI-2015 components and scoring standards; Table S2: Associations between healthy lifestyle and all-cause mortality in participants with MAFLD by gender, age, and ethnicity; Table S3: Associations of healthy lifestyle and cause-specific mortality in participants with and without MAFLD; Table S4: Associations between healthy lifestyle and all-cause mortality in participants with and without NAFLD; Table S5: Associations between healthy lifestyle and all-cause mortality in NAFLD participants with and without advanced fibrosis; Table S6: Associations between healthy lifestyle and all-cause mortality in NAFLD participants with and without elevated liver enzyme; Table S7: Associations between individual healthy lifestyle and all-cause mortality in participants with and without MAFLD or NAFLD; Table S8: Associations between individual healthy lifestyle and all-cause mortality in MAFLD participants with and without advanced fibrosis; Table S9: Associations between individual healthy lifestyle and all-cause mortality in NAFLD participants with and without advanced fibrosis.

## List of Abbreviations

MAFLD, metabolic associated fatty liver disease; NAFLD, non-alcoholic fatty liver disease; US, United States; NHANES, National Health Nutrition Examination Survey; HEI, healthy eating index; FLI, fatty liver index; BMI, body mass index; WC, waist circumference; HDL, high-density lipoprotein; HOMA-IR, homeostasis model assessment of insulin resistance; CRP, C-creative protein; NFS, NAFLD fibrosis score; FIB-4, fibrosis-4; ALT, alanine aminotransferase; GGT, γ-glutamyl transferase; NDI, National Death Index; UCOD_113, Underlying Causes of Death 113; ICD-10, International Classification of Diseases; HR, hazards ratio; CI, confidence interval; RCT, randomized controlled trial; CAP, controlled attenuation parameter; APRI, aspartate aminotransferase/platelet ratio index.
