# Supplementary material for: Associations between Healthy Lifestyle and All-Cause Mortality in Individuals with Metabolic Associated Fatty Liver Disease
Source: Nutrients. 2022 Oct 11;14(20):4222. doi: 10.3390/nu14204222 (PMC9609442; doi:10.3390/nu14204222)
Supplement: Supplementary file 1 [file nutrients-14-04222-s001.zip › nutrients-1941890-supplementary.pdf]

Supplementary Material

Table of content

Table S1. HEI-2015 components and scoring standards 2
Table S2. Associations between healthy lifestyle and all-cause mortality in participants with MAFLD by gender, age, and ethnicity 3
Table S3. Associations of healthy lifestyle and cause-specific mortality in participants with and without MAFLD 5
Table S4. Associations between healthy lifestyle and all-cause mortality in participants with and without NAFLD 6
Table S5. Associations between healthy lifestyle and all-cause mortality in NAFLD participants with and without advanced fibrosis 7
Table S6. Associations between healthy lifestyle and all-cause mortality in NAFLD participants with and without elevated liver enzyme 9
Table S7. Associations between individual healthy lifestyle and all-cause mortality in participants with and without MAFLD or NAFLD 10
Table S8. Associations between individual healthy lifestyle and all-cause mortality in MAFLD participants with and without advanced fibrosis 11
Table S9. Associations between individual healthy lifestyle and all-cause mortality in NAFLD participants with and without advanced fibrosis 13

Table S1. HEI-2015 components and scoring standards[1]

Component	Maximum points	Standards for maximum score	Standards for minimum score of zero
**Adequacy**			
Total fruits[2]	5	≥0.8 cup equiv. per 1000 kcal	No fruits
Whole fruits[3]	5	≥0.4 cup equiv. per 1000 kcal	No whole fruits
Total vegetables[4]	5	≥1.1 cup equiv. per 1000 kcal	No vegetables
Greens and beans[4]	5	≥0.2 cup equiv. per 1000 kcal	No dark green vegetables or legumes
Whole grains	10	≥1.5 oz equiv. per 1000 kcal	No whole grains
Diary[5]	10	≥1.3 cup equiv. per 1000 kcal	No diary
Total protein foods[6]	5	≥2.5 oz equiv. per 1000 kcal	No protein foods
Seafood and plant proteins[6, 7]	5	≥0.8 oz equiv. per 1000 kcal	No seafood or plant proteins
Fatty acids[8]	10	(PUFAs + MUFAs)/SFAs≥2.5	(PUFAs + MUFAs)/SFAs≤1.2
**Moderation**			
Refined grains	10	≤1.8 oz equiv. per 1000 kcal	≥4.3 oz equiv. per 1000 kcal
Sodium	10	≤1.1 gram per 1000 kcal	≥2.0 grams per 1000 kcal
Added sugars	10	≤6.5% of energy	≥26% of energy
Saturated fats	10	≤8% of energy	≥16% of energy

1: Intakes between the minimum and maximum standards are scored proportionately. The total HEI score is the sum of the adequacy components (i.e., foods to eat more of for good health) and moderation components (i.e., foods to limit for good health).

2: Including 100% fruit juice.

3: Including all forms except juice.

4: Including legumes (beans and peas).

5: Including all milk products, such as fluid milk, yogurt, and cheese, and fortified soy beverages.

6: Including legumes (beans and peas).

7: Including seafood, nuts, seeds, soy products (other than beverages), and legumes (beans and peas).

8: Ratio of poly- and monounsaturated fatty acids (PUFAs and MUFAs) to saturated fatty acids (SFAs).

Table S2. Associations between healthy lifestyle and all-cause mortality in participants with MAFLD by gender, age, and ethnicity

	No. death/no. participants	Model 1	Model 2
		HR (95%CI)	HR (95%CI)
**Male (n=1484)**			
Score 0/1	181/494	1.00	1.00
Score 2	169/566	0.65 (0.52, 0.82)	0.67 (0.53, 0.83)
Score 3/4	109/424	0.55 (0.42, 0.73)	0.59 (0.44, 0.78)
Per 1-unit increase		*0.74 (0.65, 0.83)*	*0.75 (0.66, 0.85)*
**Female (n=1132)**			
Score 0/1	88/259	1.00	1.00
Score 2	126/471	0.71 (0.48, 1.04)	0.82 (0.52, 1.28)
Score 3/4	113/402	0.63 (0.44, 0.90)	0.72 (0.48, 1.08)
Per 1-unit increase		*0.80 (0.69, 0.94)*	*0.85 (0.72, 1.01)*
P-value for interaction		0.54	0.59
**Age ≥50 (n=1630)**			
Score 0/1	226/450	1.00	1.00
Score 2	270/673	0.68 (0.54, 0.86)	0.70 (0.56, 0.89)
Score 3/4	211/507	0.67 (0.53, 0.87)	0.70 (0.54, 0.90)
Per 1-unit increase		*0.80 (0.72, 0.90)*	*0.84 (0.75, 0.94)*
**Age <50 (n=986)**			
Score 0/1	43/303	1.00	1.00
Score 2	25/364	0.51 (0.25, 1.06)	0.55 (0.30, 1.00)
Score 3/4	11/319	0.13 (0.06, 0.32)	0.10 (0.03, 0.31)
Per 1-unit increase		*0.43 (0.30, 0.60)*	*0.40 (0.29, 0.55)*
P-value for interaction		<0.001	<0.001
**Non-Hispanic white (n=1310)**			
Score 0/1	162/411	1.00	1.00
Score 2	180/516	0.64 (0.49, 0.82)	0.67 (0.52, 0.87)
Score 3/4	128/383	0.56 (0.42, 0.73)	0.58 (0.44, 0.76)
Per 1-unit increase		*0.74 (0.65, 0.84)*	*0.75 (0.66, 0.85)*
**Other races (n=1306)**			
Score 0/1	107/342	1.00	1.00
Score 2	115/521	0.67 (0.40, 1.14)	0.66 (0.41, 1.06)
Score 3/4	94/443	0.56 (0.37, 0.85)	0.56 (0.36, 0.88)
Per 1-unit increase		*0.78 (0.65, 0.92)*	*0.78 (0.64, 0.94)*
P-value for interaction		0.71	0.91

Survey-weight adjusted multivariable Cox proportional models were used in this analysis.

Model 1 was adjusted for age, gender, ethnicity, education, and marital status (exclude stratification variables for gender and ethnicity subgroups).

Model 2 was adjusted for BMI, WC, CRP, TG, HDL, hypertension, and diabetes in addition to model 1.

CI, confidence interval; HR, hazards ratio.

Table S3. Associations of healthy lifestyle and cause-specific mortality in participants with and without MAFLD

	No. CVD death/no. participants	Model 1	Model 2	No. cancer death/no. participants	Model 1	Model 2
		HR (95%CI)	HR (95%CI)		HR (95%CI)	HR (95%CI)
**Non-MAFLD**						
Score 0/1	88/1217	1.00	1.00	78/1217	1.00	1.00
Score 2	88/1939	0.71 (0.47, 1.06)	0.77 (0.50, 1.16)	72/1939	0.49 (0.30, 0.79)	0.52 (0.32, 0.83)
Score 3/4	101/2140	0.71 (0.48, 1.05)	0.78 (0.52, 1.17)	76/2140	0.47 (0.31, 0.70)	0.50 (0.33, 0.74)
Per 1-unit increase		*0.83 (0.71, 0.97)*	*0.86 (0.73, 1.01)*		*0.73 (0.61, 0.87)*	*0.75 (0.63, 0.89)*
**MAFLD**						
Score 0/1	59/753	1.00	1.00	71/753	1.00	1.00
Score 2	98/1037	1.03 (0.68, 1.56)	1.07 (0.70, 1.64)	49/1037	0.45 (0.28, 0.74)	0.45 (0.27, 0.73)
Score 3/4	55/826	0.70 (0.46, 1.07)	0.75 (0.49, 1.16)	49/826	0.54 (0.30, 0.97)	0.52 (0.29, 0.90)
Per 1-unit increase		*0.81 (0.67, 0.97)*	*0.83 (0.68, 1.00)*		*0.71 (0.52, 0.97)*	*0.69 (0.52, 0.93)*
P-value for interaction		0.89	0.97		0.73	0.87

Survey-weight adjusted multivariable Cox proportional models were used in this analysis.

Model 1 was adjusted for age, gender, ethnicity, education, and marital status.

Model 2 was adjusted for BMI, WC, CRP, TG, HDL, hypertension, and diabetes in addition to model 1.

CI, confidence interval; HR, hazards ratio; MAFLD, metabolic associated fatty liver disease.

Table S4. Associations between healthy lifestyle and all-cause mortality in participants with and without NAFLD

	No. death/no. participants	Model 1	Model 2
		HR (95%CI)	HR (95%CI)
**Non-NAFLD (n=5466)**			
Score 0/1	348/1332	1.00	1.00
Score 2	382/1986	0.71 (0.59, 0.85)	0.75 (0.63, 0.90)
Score 3	314/1649	0.59 (0.50, 0.70)	0.64 (0.54, 0.75)
Score 4	59/499	0.35 (0.26, 0.46)	0.39 (0.30, 0.51)
Per 1-unit increase		*0.75 (0.70, 0.80)*	*0.78 (0.74, 0.83)*
**NAFLD (n=2446)**			
Score 0/1	222/638	1.00	1.00
Score 2	278/990	0.67 (0.56, 0.83)	0.69 (0.55, 0.87)
Score 3	193/684	0.62 (0.49, 0.79)	0.65 (0.50, 0.83)
Score 4	24/134	0.28 (0.18, 0.45)	0.30 (0.19, 0.49)
Per 1-unit increase		*0.75 (0.67, 0.83)*	*0.76 (0.68, 0.85)*
P value for interaction		0.88	0.92

Survey-weight adjusted multivariable Cox proportional models were used in this analysis.

Model 1 was adjusted for age, gender, ethnicity, education, and marital status.

Model 2 was adjusted for BMI, WC, CRP, TG, HDL, hypertension, and diabetes in addition to model 1.

CI, confidence interval; HR, hazards ratio; NAFLD, non-alcoholic fatty liver index; MAFLD, metabolic associated fatty liver disease.

Table S5. Associations between healthy lifestyle and all-cause mortality in NAFLD participants with and without advanced fibrosis

	No. death/no. participants	Model 1	Model 2
		HR (95%CI)	HR (95%CI)
**NFS (n=2407)**			
**Low NFS (n=1266)**			
Score 0/1	72/338	1.00	1.00
Score 2	54/497	0.43 (0.23, 0.79)	0.38 (0.23, 0.64)
Score 3	49/365	0.46 (0.27, 0.76)	0.37 (0.23, 0.61)
Score 4	7/66	0.25 (0.12, 0.52)	0.23 (0.11, 0.49)
Per 1-unit increase		*0.64 (0.48, 0.84)*	*0.59 (0.46, 0.77)*
**High NFS (n=1141)**			
Score 0/1	146/295	1.00	1.00
Score 2	217/477	0.84 (0.65, 1.08)	0.89 (0.68, 1.15)
Score 3	137/304	0.74 (0.56, 0.98)	0.79 (0.58, 1.07)
Score 4	16/65	0.33 (0.19, 0.58)	0.38 (0.22, 0.70)
Per 1-unit increase		*0.81 (0.73, 0.90)*	*0.84 (0.74, 0.94)*
P value for interaction		0.04	0.01
**FIB-4 (n=2434)**			
**Low FIB-4 (n=1680)**			
Score 0/1	113/469	1.00	1.00
Score 2	111/662	0.63 (0.45, 0.89)	0.66 (0.47, 0.93)
Score 3	72/466	0.52 (0.35, 0.78)	0.51 (0.34, 0.79)
Score 4	10/83	0.26 (0.14, 0.50)	0.23 (0.11, 0.49)
Per 1-unit increase		*0.68 (0.57, 0.82)*	*0.67 (0.56, 0.81)*
**High FIB-4 (n=754)**			
Score 0/1	109/169	1.00	1.00
Score 2	167/322	0.68 (0.49, 0.95)	0.70 (0.50, 0.97)
Score 3	121/214	0.69 (0.48, 0.99)	0.72 (0.50, 1.04)
Score 4	14/49	0.32 (0.16, 0.66)	0.38 (0.19, 0.76)
Per 1-unit increase		*0.80 (0.69, 0.94)*	*0.83 (0.71, 0.96)*
P value for interaction		0.08	0.03
**Forn's score (n=2438)**			
**Low Forn's score (n=1322)**			
Score 0/1	61/355	1.00	1.00
Score 2	61/520	0.70 (0.43, 1.12)	0.62 (0.38, 0.99)
Score 3	51/382	0.56 (0.32, 0.98)	0.49 (0.27, 0.88)
Score 4	4/65	0.17 (0.06, 0.46)	0.14 (0.05, 0.40)
Per 1-unit increase		*0.70 (0.54, 0.90)*	*0.65 (0.50, 0.84)*
**High Forn's score (n=1116)**			

Score 0/1	161/283	1.00	1.00
Score 2	217/469	0.65 (0.51, 0.82)	0.67 (0.53, 0.86)
Score 3	142/297	0.62 (0.47, 0.81)	0.67 (0.50, 0.89)
Score 4	20/67	0.32 (0.19, 0.54)	0.38 (0.23, 0.64)
Per 1-unit increase		*0.76 (0.68, 0.86)*	*0.80 (0.71, 0.89)*
P value for interaction		0.26	0.08

Survey-weight adjusted multivariable Cox proportional models were used in this analysis.

Model 1 was adjusted for age, gender, ethnicity, education, and marital status.

Model 2 was adjusted for BMI, WC, CRP, TG, HDL, hypertension, and diabetes in addition to model 1.

Cut-off values for fibrosis biomarkers: NFS, -1.455; FIB-4, 1.30; Forn's score, 4.21

CI, confidence interval; FIB-4, fibrosis-4; HR, hazards ratio; NFS, NAFLD fibrosis score; MAFLD, metabolic associated fatty liver disease.

Table S6. Associations between healthy lifestyle and all-cause mortality in NAFLD participants with and without elevated liver enzyme

	No. death/no. participants	Model 1	Model 2
		HR (95%CI)	HR (95%CI)
**ALT (n=2441)**			
**Normal ALT (n=1847)**			
Score 0/1	187/477	1.00	1.00
Score 2	250/772	0.75 (0.60, 0.94)	0.77 (0.61, 0.97)
Score 3	163/499	0.66 (0.51, 0.85)	0.70 (0.53, 0.92)
Score 4	19/99	0.26 (0.17, 0.38)	0.28 (0.19, 0.43)
Per 1-unit increase		*0.75 (0.68, 0.83)*	*0.77 (0.68, 0.86)*
**Elevated ALT (n=594)**			
Score 0/1	35/161	1.00	1.00
Score 2	28/213	0.43 (0.22, 0.85)	0.42 (0.21, 0.85)
Score 3	30/185	0.47 (0.24, 0.90)	0.41 (0.20, 0.84)
Score 4	5/35	0.56 (0.18, 1.74)	0.59 (0.19, 1.88)
Per 1-unit increase		*0.76 (0.55. 1.05)*	*0.72 (0.51, 1.02)*
P value for interaction		0.83	0.95
**GGT (n=2446)**			
**Normal GGT (n=1548)**			
Score 0/1	135/377	1.00	1.00
Score 2	185/642	0.69 (0.52, 0.93)	0.74 (0.54, 1.02)
Score 3	131/440	0.56 (0.42, 0.76)	0.63 (0.46, 0.87)
Score 4	14/89	0.22 (0.11, 0.43)	0.28 (0.14, 0.54)
Per 1-unit increase		*0.70 (0.62, 0.80)*	*0.74 (0.64, 0.85)*
**Elevated GGT (n=898)**			
Score 0/1	87/261	1.00	1.00
Score 2	93/348	0.62 (0.42, 0.90)	0.59 (0.41, 0.84)
Score 3	62/244	0.71 (0.48, 1.05)	0.68 (0.46, 1.00)
Score 4	10/45	0.50 (0.27, 0.91)	0.49 (0.25, 0.99)
Per 1-unit increase		*0.83 (0.71, 0.98)*	*0.82 (0.69, 0.97)*
P value for interaction		0.22	0.25

Survey-weight adjusted multivariable Cox proportional models were used in this analysis.

Model 1 was adjusted for age, gender, ethnicity, education, and marital status.

Model 2 was adjusted for BMI, WC, CRP, TG, HDL, hypertension, and diabetes in addition to model 1.

Cut-off values for liver enzymes: ALT, 40/31 IU/L for men/women; GGT, 51/33 IU/L for men/women

ALT, alanine aminotransferase; AST, aspartate aminotransferase; CI, confidence interval; GGT, γ-glutamyl transferase; HR, hazards ratio; MAFLD, metabolic associated fatty liver disease.

Table S7. Associations between individual healthy lifestyle and all-cause mortality in participants with and without MAFLD or NAFLD

	Model 1	Model 2
	HR (95%CI)	HR (95%CI)
**MAFLD**		
**Non-MAFLD (n=5296)**		
Never smoking	0.79 (0.67, 0.94)	0.81 (0.69, 0.96)
No heavy alcohol drinking	0.58 (0.44, 0.77)	0.65 (0.48, 0.88)
Top tertile of LTPA	0.83 (0.68, 1.02)	0.84 (0.69, 1.02)
Top two quintiles of HEI-2015 score	0.71 (0.61, 0.82)	0.71 (0.61, 0.83)
**MAFLD (n=2616)**		
Never smoking	0.69 (0.57, 0.84)	0.72 (0.59, 0.88)
No heavy alcohol drinking	0.81 (0.59, 1.12)	0.81 (0.56, 1.16)
Top tertile of LTPA	0.92 (0.76, 1.12)	0.91 (0.75, 1.10)
Top two quintiles of HEI-2015 score	0.63 (0.52, 0.77)	0.65 (0.53, 0.80)
**NAFLD**		
**Non-NAFLD (n=5466)**		
Never smoking	0.78 (0.67, 0.91)	0.81 (0.69, 0.94)
No heavy alcohol drinking	0.61 (0.47, 0.80)	0.70 (0.53, 0.92)
Top tertile of LTPA	0.86 (0.71, 1.03)	0.86 (0.72, 1.03)
Top two quintiles of HEI-2015 score	0.70 (0.61, 0.80)	0.72 (0.62, 0.83)
**NAFLD (n=2446)**		
Never smoking	0.69 (0.57, 0.82)	0.72 (0.60, 0.86)
No heavy alcohol drinking	0.90 (0.52, 1.57)	0.82 (0.46, 1.48)
Top tertile of LTPA	0.88 (0.72, 1.09)	0.89 (0.72, 1.09)
Top two quintiles of HEI-2015 score	0.63 (0.50, 0.78)	0.64 (0.51, 0.81)

Survey-weight adjusted multivariable Cox proportional models were used in this analysis.

Model 1 was adjusted for age, gender, ethnicity, education, and marital status.

Model 2 was adjusted for BMI, WC, CRP, TG, HDL, hypertension, and diabetes in addition to model 1.

CI, confidence interval; HEI, healthy eating index; HR, hazards ratio; LTPA, leisure-time physical activity; NAFLD, non-alcoholic fatty liver index; MAFLD, metabolic associated fatty liver disease.

Table S8. Associations between individual healthy lifestyle and all-cause mortality in MAFLD participants with and without advanced fibrosis

	Model 1	Model 2
	HR (95%CI)	HR (95%CI)
**NFS (n=2407)**		
**Low NFS (n=1266)**		
Never smoking	0.52 (0.34, 0.79)	0.44 (0.30, 0.65)
No heavy alcohol drinking	0.81 (0.42, 1.55)	0.87 (0.43, 1.76)
Top tertile of LTPA	0.81 (0.57, 1.17)	0.81 (0.56, 1.17)
Top two quintiles of HEI-2015 score	0.57 (0.36, 0.90)	0.61 (0.38, 0.97)
**High NFS (n=1228)**		
Never smoking	0.79 (0.63, 0.99)	0.84 (0.67, 1.06)
No heavy alcohol drinking	0.87 (0.56, 1.34)	0.84 (0.50, 1.39)
Top tertile of LTPA	1.00 (0.81, 1.24)	1.00 (0.80, 1.24)
Top two quintiles of HEI-2015 score	0.64 (0.51, 0.81)	0.67 (0.54, 0.84)
**FIB-4 (n=2604)**		
**Low FIB-4 (n=1773)**		
Never smoking	0.70 (0.50, 0.97)	0.72 (0.51, 0.97)
No heavy alcohol drinking	0.65 (0.42, 1.01)	0.65 (0.42, 1.00)
Top tertile of LTPA	0.94 (0.68, 1.31)	0.88 (0.63, 1.23)
Top two quintiles of HEI-2015 score	0.53 (0.38, 0.73)	0.55 (0.39, 0.77)
**High FIB-4 (n=831)**		
Never smoking	0.69 (0.52, 0.91)	0.73 (0.56, 0.97)
No heavy alcohol drinking	1.02 (0.65, 1.61)	1.04 (0.62, 1.74)
Top tertile of LTPA	0.92 (0.71, 1.20)	0.96 (0.72, 1.28)
Top two quintiles of HEI-2015 score	0.69 (0.51, 0.93)	0.69 (0.51, 0.92)
**Forn’s score (n=2608)**		
**Low Forn’s score (n=1384)**		
Never smoking	0.74 (0.49, 1.13)	0.65 (0.42, 0.98)
No heavy alcohol drinking	0.62 (0.30, 1.29)	0.81 (0.40, 1.64)
Top tertile of LTPA	0.80 (0.53, 1.20)	0.72 (0.48, 1.07)
Top two quintiles of HEI-2015 score	0.39 (0.23, 0.66)	0.44 (0.25, 0.78)
**High Forn’s score (n=1224)**		
Never smoking	0.69 (0.55, 0.88)	0.75 (0.60, 0.94)
No heavy alcohol drinking	0.94 (0.58, 1.53)	0.92 (0.54, 1.55)
Top tertile of LTPA	0.94 (0.76, 1.17)	0.95 (0.77, 1.18)
Top two quintiles of HEI-2015 score	0.77 (0.62, 0.95)	0.78 (0.63, 0.97)

Survey-weight adjusted multivariable Cox proportional models were used in this analysis.

Model 1 was adjusted for age, gender, ethnicity, education, and marital status.

Model 2 was adjusted for BMI, WC, CRP, TG, HDL, hypertension, and diabetes in addition to model 1.

Cut-off values for fibrosis biomarkers: NFS, -1.455; FIB-4, 1.30; Forn's score, 4.21

CI, confidence interval; FIB-4, fibrosis-4; HEI, healthy eating index; HR, hazards ratio; LTPA, leisure-time physical activity; NFS, NAFLD fibrosis score; MAFLD, metabolic associated fatty liver disease.

Table S9. Associations between individual healthy lifestyle and all-cause mortality in NAFLD participants with and without advanced fibrosis

	Model 1	Model 2
	HR (95%CI)	HR (95%CI)
**NFS (n=2576)**		
**Low NFS (n=1348)**		
Never smoking	0.54 (0.35, 0.82)	0.44 (0.29, 0.66)
No heavy alcohol drinking	0.69 (0.26, 1.84)	0.66 (0.24, 1.84)
Top tertile of LTPA	0.81 (0.56, 1.16)	0.77 (0.54, 1.10)
Top two quintiles of HEI-2015 score	0.58 (0.36, 0.93)	0.60 (0.36, 0.97)
**High NFS (n=1141)**		
Never smoking	0.77 (0.62, 0.97)	0.82 (0.66, 1.03)
No heavy alcohol drinking	1.45 (0.65, 3.23)	1.22 (0.49, 3.05)
Top tertile of LTPA	0.96 (0.77, 1.21)	0.98 (0.78, 1.24)
Top two quintiles of HEI-2015 score	0.63 (0.49, 0.81)	0.65 (0.50, 0.84)
**FIB-4 (n=2434)**		
**Low FIB-4 (n=1680)**		
Never smoking	0.71 (0.52, 0.96)	0.72 (0.53, 0.97)
No heavy alcohol drinking	0.57 (0.28, 1.16)	0.51 (0.25, 1.05)
Top tertile of LTPA	0.86 (0.62, 1.19)	0.81 (0.57, 1.14)
Top two quintiles of HEI-2015 score	0.50 (0.35, 0.71)	0.51 (0.35, 0.75)
**High FIB-4 (n=754)**		
Never smoking	0.67 (0.50, 0.90)	0.71 (0.53, 0.95)
No heavy alcohol drinking	2.08 (1.06, 4.11)	2.06 (0.91, 4.69)
Top tertile of LTPA	0.93 (0.72, 1.20)	0.98 (0.74, 1.29)
Top two quintiles of HEI-2015 score	0.70 (0.50, 0.98)	0.70 (0.50, 0.96)
**Forn's score (n=2438)**		
**Low Forn's score (n=1322)**		
Never smoking	0.83 (0.55, 1.25)	0.67 (0.44, 1.02)
No heavy alcohol drinking	0.71 (0.23, 2.12)	0.73 (0.23, 2.36)
Top tertile of LTPA	0.78 (0.52, 1.17)	0.73 (0.48, 1.10)
Top two quintiles of HEI-2015 score	0.44 (0.26, 0.75)	0.49 (0.27, 0.89)
**High Forn's score (n=1116)**		
Never smoking	0.64 (0.52, 0.79)	0.70 (0.57, 0.86)
No heavy alcohol drinking	1.09 (0.56, 2.11)	0.91 (0.44, 1.88)
Top tertile of LTPA	0.90 (0.71, 1.13)	0.92 (0.73, 1.16)
Top two quintiles of HEI-2015 score	0.72 (0.57, 0.91)	0.73 (0.57, 0.93)

Survey-weight adjusted multivariable Cox proportional models were used in this analysis.

Model 1 was adjusted for age, gender, ethnicity, education, and marital status.

Model 2 was adjusted for BMI, WC, CRP, TG, HDL, hypertension, and diabetes in addition to model 1.

Cut-off values for fibrosis biomarkers: NFS, -1.455; FIB-4, 1.30; Forn's score, 4.21

CI, confidence interval; FIB-4, fibrosis-4; HEI, healthy eating index; HR, hazards ratio; LTPA, leisure-time physical activity; NFS, NAFLD fibrosis score
